# A case of intraductal papillary neoplasm of the bile duct suspected to be of peribiliary glands origin

**DOI:** 10.1002/deo2.70001

**Published:** 2024-08-21

**Authors:** Takuya Ogiso, Hirotaka Suzuki, Hiroshi Matsubara, Takehito Naito, Masahiro Yamada, Hideko Yamamoto, Shun Hattori, Taro Aoba, Yoshifumi Arai, Fumihiro Urano

**Affiliations:** ^1^ Department of Gastroenterology Chutoen General Medical Center Shizuoka Japan; ^2^ Department of Gastroenterology Toyohashi Municipal Hospital Aichi Japan; ^3^ Department of General Surgery Toyohashi Municipal Hospital Aichi Japan; ^4^ Department of Pathological Diagnosis Toyohashi Municipal Hospital Aichi Japan

**Keywords:** bile duct, intraductal papillary neoplasm of the bile duct, mucobilia, peribiliary glands, peroral cholangioscopy

## Abstract

Peribiliary glands are complex lobular structures containing mucus and serous glands, distributed along the extrahepatic and intrahepatic bile ducts. In this report, we describe a case of intraductal papillary neoplasm of the bile duct suspected to be of peribiliary glands origin. The patient was an 80‐year‐old man who was referred to our hospital for a hepatic mass. On further examination, a 38 × 34 mm cystic lesion with papillary growth was found in S1/4. Because the lesion was extensively bordered by both hepatic ducts and the connection was unclear, it was difficult to determine the extent of hepatic resection. To confirm the location, a peroral cholangioscopy was performed. The connection with the cyst was detected in the right hepatic duct and a villous tumor mucosa protruded through the conduit lumen. Since we found that the lesion communicated with the right hepatic duct, a right hepatectomy was subsequently performed. The postoperative pathological diagnosis was an intraductal papillary neoplasm of the blie duct with associated invasive carcinoma. The postoperative course was good, and the patient experienced no recurrence.

## INTRODUCTION

Peribiliary glands exist around the large bile ducts and secrete mucinous glycoprotein.^1^ Peribiliary glands contain stem cells that can differentiate into the biliary and pancreatic cells,^2^ and several studies indicated that some intraductal papillary neoplasm of the bile ducts (IPNBs) were derived from peribiliary glands.^3^, ^4^, ^5^ IPNB is pathologically defined as a biliary neoplasm with papillary growth and includes low‐grade dysplasia to invasive carcinoma. It has delicate fibrovascular stalks and papillary epithelium covers the surface. Most IPNBs arise from large bile ducts, and dilated bile ducts filled with a papillary neoplasm are the diagnostic feature. IPNB is considered a precancerous condition of cholangiocarcinoma; therefore, surgery is generally performed. In the present case, the IPNB bordered both hepatic ducts extensively, and it was necessary to confirm the bile duct connection with the IPNB to determine accurate hepatic resection. We report a case of IPNB suspected to be of peribiliary glands origin for which we were able to obtain the accurate localization and the preoperative pathological diagnosis using peroral cholangioscopy (POCS).

## CASE REPORT

An 80‐year‐old Japanese man without any symptoms or history was referred to our hospital for a hepatic mass. He was 158 cm in height and 62 kg in weight and had no jaundice or abdominal tenderness. He had never smoked or consumed alcohol. Blood tests at the first visit showed no elevation in hepatobiliary enzymes or inflammatory response markers. The results of tumor markers were as follows: CEA, 0.9 ng/mL; CA19‐9, 43.8 U/mL; AFP,2.6 ng/mL; PIVKA2, 3 mAU/mL; DUPAN2, <25 U/mL; and Span‐1, 13.4 U/mL.

**FIGURE 1:**
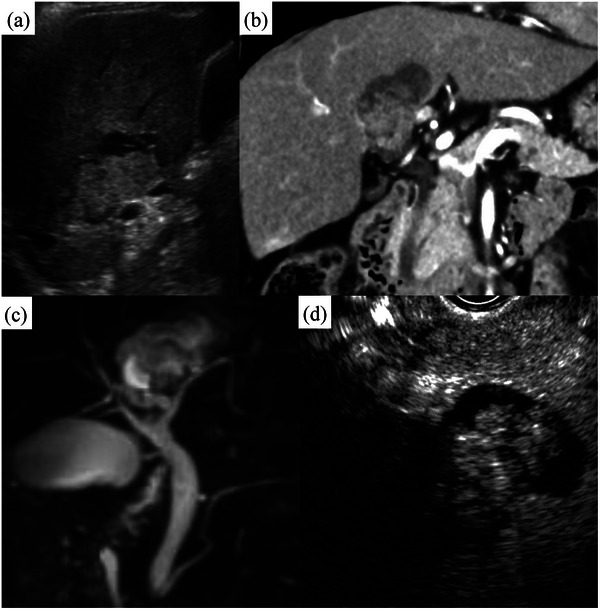
(a) Transabdominal ultrasound: A 38 × 30 mm cystic lesion with a hyperechoic lobulated solid component was observed in S1/4. There were no findings suggestive of necrosis or hemorrhage, and the margin was clear. (b) Computed tomography: A cystic lesion measuring 38 × 34 mm with papillary growth in S1/4 just above the perihilar bile duct. (c) Magnetic resonance cholangiopancreatography: The lesion was widely bordered by the left and right hepatic ducts, but the connection with the cyst was unclear. (d) Endoscopic ultrasonography: Contrast‐enhanced endoscopic ultrasonography via Sonazoid revealed deep staining in the lobulated solid component, beginning in the early phase with a prolonged contrast effect.

Transabdominal ultrasound (Figure 1a) revealed a 38 × 30 mm cystic lesion with a hyperechoic lobulated component in S1/4. There were no findings suggestive of necrosis or hemorrhage, and the margin was clear. Contrast‐enhanced computed tomography (Figure 1b) showed a cystic lesion measuring 38 × 34 mm with papillary growth in S1/4 just above the perihilar bile duct. Magnetic resonance imaging (Figure 1c) showed that the lesion was widely bordered by the left and right hepatic ducts on magnetic resonance cholangiopancreatography but the connection was unclear. Endoscopic ultrasonography (Figure 1d) revealed a cystic lesion in S1/4. Contrast enhancement via Sonazoid showed deep staining in the lobulated solid component, beginning in the early phase with a prolonged contrast effect.

**FIGURE 2:**
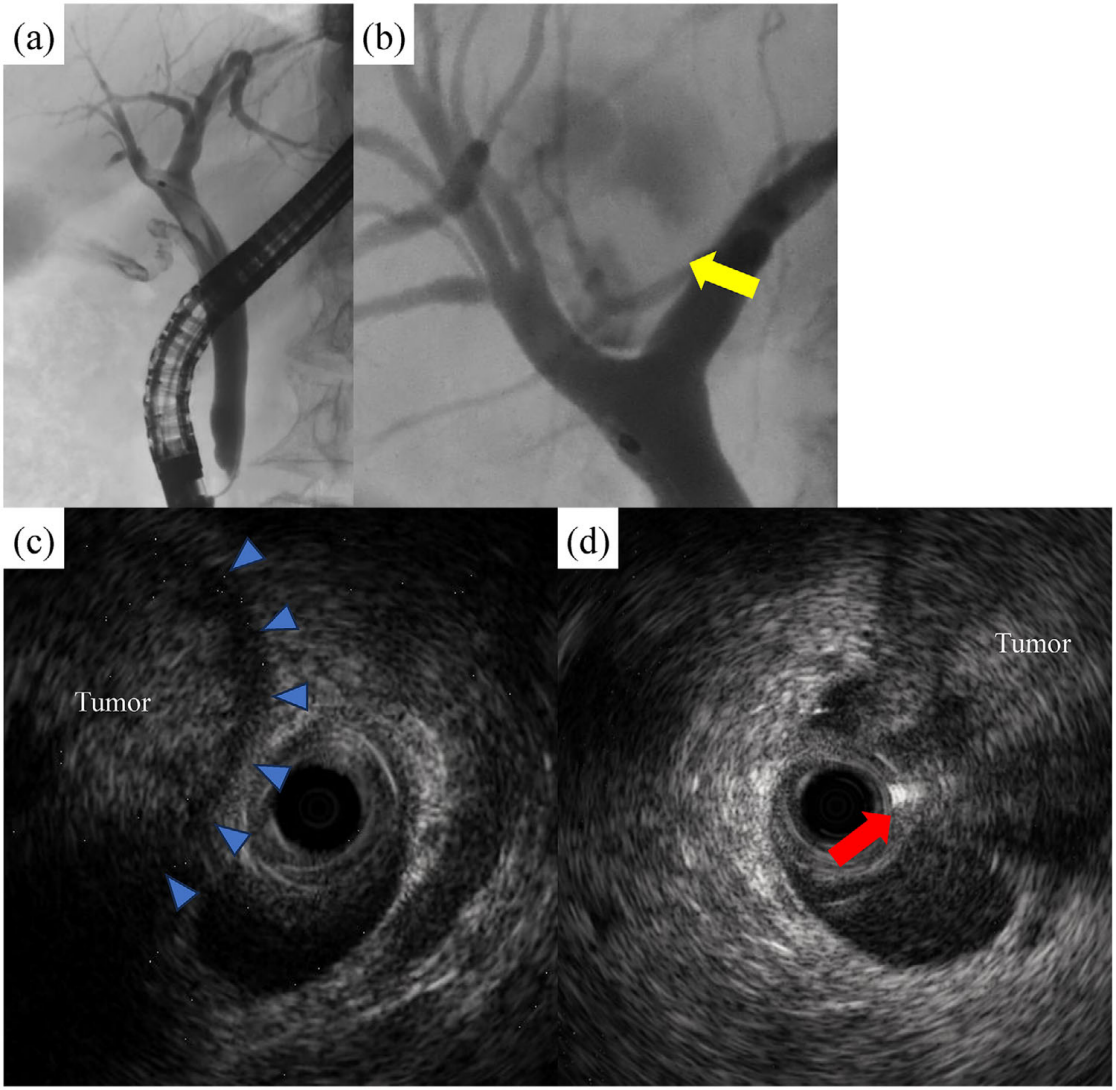
(a) A linear translucent image was observed in the common bile duct, which suggested mucus accumulation. (b) A cystic lesion with an internal irregular defect above the perihilar bile duct (yellow arrow), but accurate localization of the connection was difficult to identify via only endoscopic retrograde cholangiography. (c) The left hepatic duct was in contact with the lesion, but the layered structure of the cyst was preserved (blue arrow). (d) The right hepatic duct lost bile duct continuity, and the tumor was exposed to the bile duct lumen (red arrow).

Endoscopic retrograde cholangiopancreatography (Figure 2a,b) showed a linear translucent image in the common bile duct, which suggested mucus accumulation. We observed a cystic lesion with an internal irregular defect above the perihilar bile duct, but accurate localization was difficult to identify via only ERC. Intraductal ultrasonography (Figure 2c,d) showed that the left hepatic duct was in contact with the lesion, but the layered structure of the cyst was preserved. While, the bile duct continuity was lost in the right hepatic duct, and the tumor was exposed to the bile duct lumen.

**FIGURE 3:**
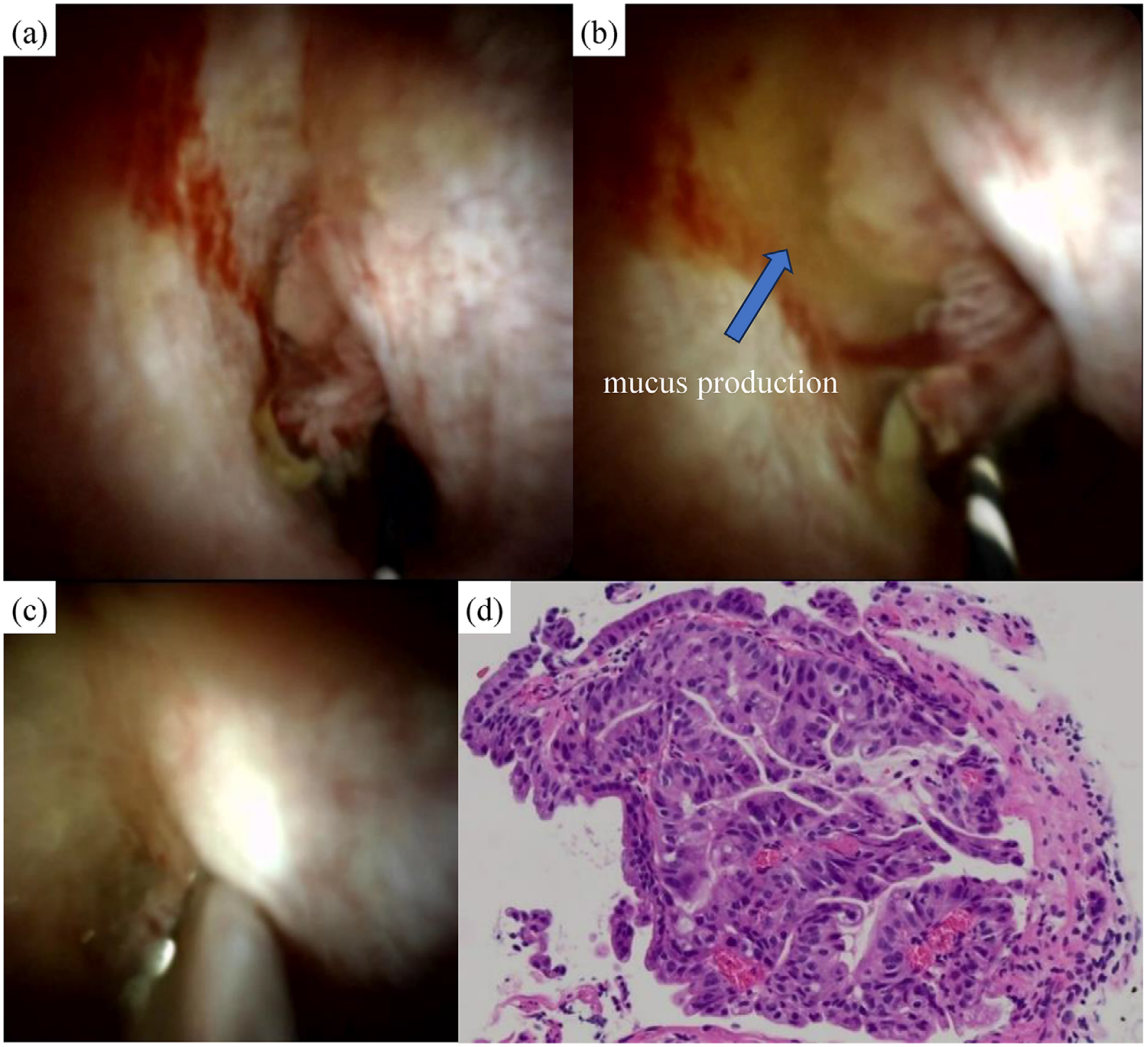
(a) The connection with the cystic lesion was located in the right hepatic duct, and villous mucosa was protruding through the conduit lumen. No superficial extension to the bile duct was observed. (b) A large amount of mucobilia drained from the cyst. (c) The tumor biopsy was performed precisely, and attributed to direct visualization. (d) Papillary epithelial neoplasm, suspicious for adenocarcinoma, was observed only in the lesion.

POCS (Figure 3a–c), using SpyGlass DS (Boston Scientific), revealed that the connection was located in the right hepatic duct, and the villous mucosa protruded through the conduit lumen. A large amount of mucobilia drained from the cyst. No superficial extension was observed. In addition, tumor biopsy and mapping biopsy were performed under direct visualization. Biopsy pathology (Figure 3d) revealed a papillary neoplasm, suspicious for adenocarcinoma, only in the lesion, while biopsies of other sites showed no neoplastic changes.

Therefore, we considered the clinical diagnosis of an IPNB communicating with the right hepatic duct. Percutaneous transhepatic portal vein embolization was performed, and the volume of the left liver lobe increased from 287 mL (37%) to 372 mL (44%). Then, a right hepatectomy with caudal lobectomy, common hepatic duct resection, and portal vein resection were performed.

**FIGURE 4:**
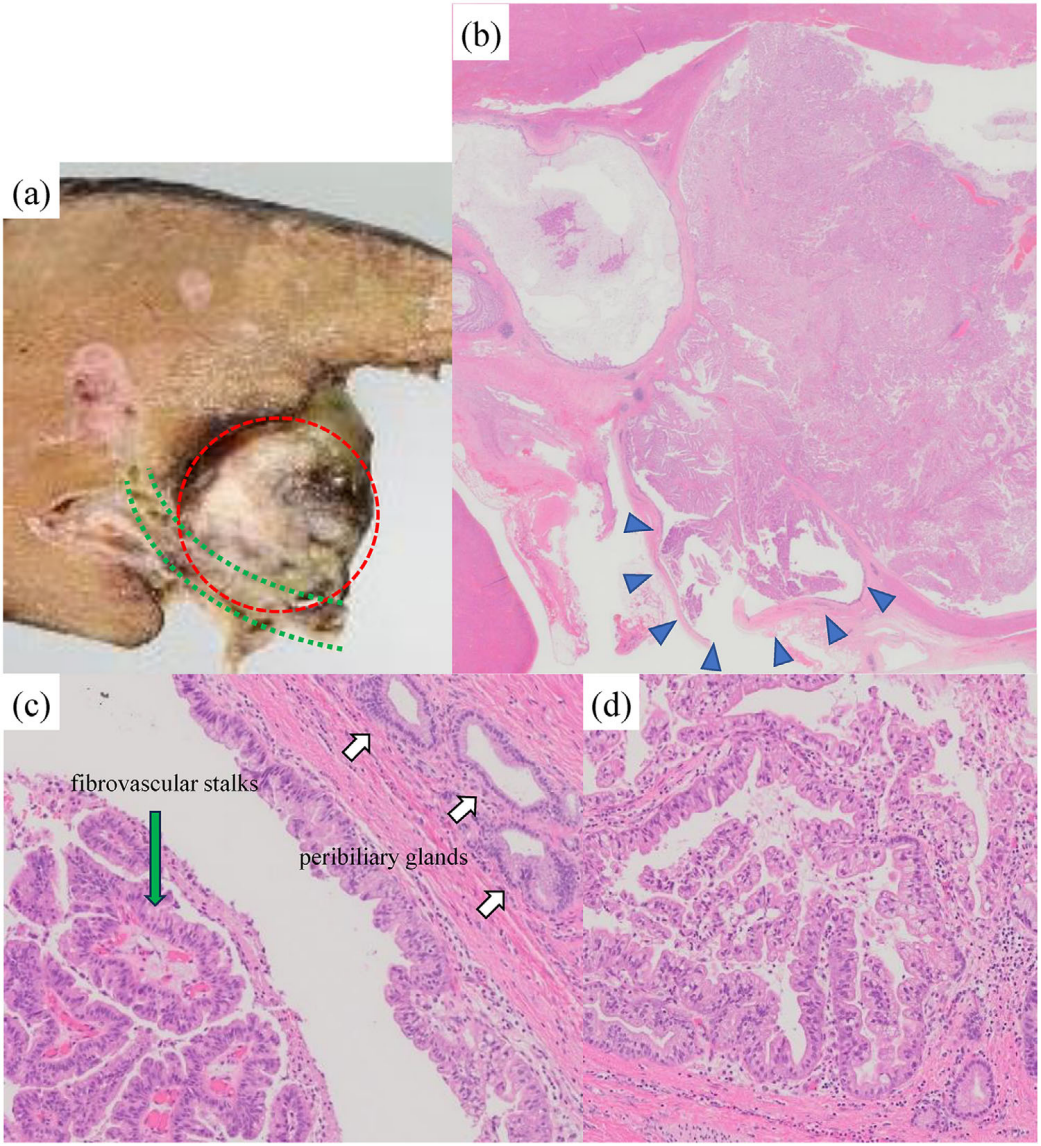
(a) The tumor (red circle) protruded into the right hepatic duct (green line). (b) The intraductal papillary neoplasm of the bile duct (IPNB) was connected to the right hepatic duct, and the papillary tumor within the cyst had spread through the conduit lumen (blue arrow). (c) Papillary growth of the neoplastic epithelium was observed on the surface of the fibrovascular stalks (green arrow). Non‐neoplastic peribiliary glands were observed near the IPNB (white arrow). (d) The tumor epithelium was papillary growth and accompanied by large and clear cells containing mucus, suggesting gastric‐type IPNB. Low‐grade and high‐grade dysplasia were also observed within the IPNB.

The postoperative pathology (Figure 4) confirmed that the lesion was connected to the right hepatic duct. No mucosal changes were observed in the right hepatic duct itself, there were non‐neoplastic peribiliary glands nearby, and there was no distal bile duct dilatation continuous from the cyst. The IPNB consisted of the tumor epithelium lining the cyst and the papillary mucosa in the lumen. The tumor epithelium was accompanied by large, clear cells and papillary growth on the surface of fibrovascular stalks, and mucus production was observed. These findings suggested gastric type IPNB. Low‐grade and high‐grade dysplasia were also observed within the IPNB, and a tiny part of the lesion invaded the liver beyond the cyst. The tubular structure was maintained, and the invaded area was well‐differentiated adenocarcinoma. The final pathological diagnosis was IPNB with associated invasive carcinoma, suspected to be of peribiliary glands origin. The patient's postoperative course was good, and there has been no recurrence for 3 years.

## DISCUSSION

According to the 2019 WHO Classification of Tumors (Digestive System) 5th edition, IPNB is defined as a premalignant tumor with papillary growth and is considered an intraepithelial neoplasia of the bile duct. In addition, IPNB is classified as type 1 or 2, which was suggested by a Japanese–Korean study group; type 1 is considered a classical IPNB and corresponds to a tumor similar to IPMN, and type 2 corresponds to conventional papillary cholangiocarcinoma. Type 1 IPNB, which is considered the counterpart of IPMN, is characterized by dilated bile ducts filled with papillary neoplasm‐producing mucobilia. Therefore, the present case was considered a type 1 IPNB. IPMNs can be classified into the following three types: main duct type, branch duct type, and mixed type. Nakanuma et al.^6^ applied this classification to type 1 IPNB and reported that IPNB originating from peribiliary glands was considered branch duct IPNB.

Generally, biliary tract tumors are speculated to be derived from the transformation of biliary stem cells.^7^ Cardinale et al.^8^ reported that peribiliary glands contain stem cells that can differentiate into biliary and pancreatic cells. These findings are supported by the fact that, embryologically, the biliary and pancreatic primordia develop from the foregut at approximately the same time and are highly plastic. The presence of stem cells within the peribiliary glands suggests that type 1 IPNBs, similar to IPMNs, may originate from the peribiliary glands, and several studies have indicated this.^3^, ^4^, ^5^ In IPNBs originating from peribiliary grands, neoplastic papillary lesions develop from the glandular epithelium, and peribiliary glands enlarge in a cystic shape while producing mucus.^9^ Therefore, unlike ordinary IPNBs, there is no dilation or neoplastic changes in the bile duct, and it is observed as a cystic lesion that communicates with the bile duct.

In the present case, the lesion was connected to the right hepatic duct through the conduit, with no mucosal changes observed in the large bile duct itself. The characteristics of the lesion were secreted mucus and cystic shape with no distal bile duct dilation. Furthermore, the lesion was located in the perihilar bile duct, an area with abundant peribiliary glands, and there were non‐neoplastic peribiliary glands nearby. Therefore, the lesion was considered an IPNB suspected to be of peribiliary glands origin.

Usually, branch duct IPNBs have a connection with bile ducts. However in the present case, the IPNB was widely bordered by both hepatic ducts, and it was difficult to identify accurate localization. Furthermore, mucobilia caused contrast defects, and we might overestimate the extent of progression via ERC. Therefore, it was difficult to determine whether right or left hepatic resection should be performed. POCS allowed direct observation of the bile ducts, and we were able to detect accurate connection sites and superficial progression.

In addition, because POCS can be used to perform biopsies under direct vision, we were able to collect tumor tissue reliably. Draganov et al.^10^ reported that the positive diagnosis rate of biopsy under direct visualization was greater than that of biopsy under fluoroscopy. Particularly in the present case, we assumed that it would be difficult to press the biopsy forceps against the tumor under fluoroscopy because a tiny portion of the IPNB protruded from the conduit. Furthermore, POCS can detect superficial progression, which may contribute to improving the diagnostic process.

In conclusion, POCS played a central role in the treatment of the IPNB and enabled an accurate surgical approach based on the location of the IPNB.

## CONFLICT OF INTEREST STATEMENT

None.

## ETHICS STATEMENT

All procedures were performed in accordance with the ethical standards of the Declaration of Helsinki and its later amendments.

## PATIENT CONSENT STATEMENT

Informed consent was obtained from the patient for the publication of this case report.
